# The initial inoculation ratio regulates bacterial coculture interactions and metabolic capacity

**DOI:** 10.1038/s41396-020-00751-7

**Published:** 2020-09-04

**Authors:** Chun-Hui Gao, Hui Cao, Peng Cai, Søren J. Sørensen

**Affiliations:** 1 State Key Laboratory of Agricultural Microbiology, College of Resources and Environment, Huazhong Agricultural University, Wuhan, 430070 China; 2 Section of Microbiology, Department of Biology, University of Copenhagen, Copenhagen, 2100 Denmark

**Keywords:** Microbial ecology, Soil microbiology

## Abstract

Coculture is an important model system in microbial ecology studies. As a key experimental parameter, the initial inoculation ratio has a crucial impact on the results of the coculture system. However, such an effect has never been investigated under multiple niche conditions. In this study, we established a simple coculture system with two model bacteria in various carbon sources and investigated the influence of initial inoculum ratios of 1:1000 to 1000:1 on community structure, function, and bacterial interaction. We found that the final ratio of the cocultures with different initial inoculum ratios differed in approximately five-sixths of the carbon sources, suggesting that the final ratio is highly dependent on the initial inoculum ratio, while the carbon source preferences of bacteria could not predict the final ratio of cocultures. Furthermore, we found that the initial ratio could regulate the metabolic capacity of the coculture, as only cocultures with initial ratios of 1:1 and 1000:1 gained high capacity on 14 specific carbon sources. The underlying reason may be that the pattern of species interaction is changed by the initial ratio. In conclusion, we showed that the initial ratio can induce emergent properties in coculture. These findings suggest that the initial ratio not only impacts the reproducibility of coculture experiments but also can influence our understanding of generic microbial ecology.

## Introduction

The application of bacterial coculture or synthetic bacterial communities is extensive in microbial ecology studies [1]. To date, coculture systems have played a fundamental role in studying species interactions [2–7], the development of multispecies biofilms [8–10], the regulation of bacterial community dynamics [11–13], and the construction of synthetic communities with specific functionalities [14–17].

To establish a coculture system, apart from choosing proper organisms and culture conditions, optimization of the initial inoculation ratio is important. The setting of the first two parameters is usually simple to determine based on study purpose and restrictions, but the choice of the initial ratio is not straightforward. In general, researchers typically inoculate different species in an initial ratio of 1:1, which is based on either the optical density at 600 nm (OD600) or cell numbers [5, 8]. However, there are some exceptions. For instance, to study multispecies biofilm formation, *Pseudomonas aeruginosa* PAO1, *P. protegens* Pf-5 and *Klebsiella pneumonia* KP-1 were inoculated in a microfluidic device with an inoculation ratio of 5:5:1 [18]. Moreover, the initial ratio in different studies can range from 1:1000 to 1000:1 [4, 19, 20]. Since the proportion of species eventually changes during cultivation, the final ratio is usually quite different from the initial ratio. For example, 24 h of cocultivation of *Stenotrophomonas rhizophila, Xanthomonas retroflexus, Ochrobactrum rhizosphaerae*, and *Paenibacillus amylolyticus* in a 1:1:1:1 inoculation ratio resulted in a final ratio of 4:900:9:15 in the formed multispecies biofilms [8]. Further investigations are required to understand whether a change in the initial inoculum ratio in a coculture system could alter the community structure.

Furthermore, few studies have revealed that the initial ratio can modulate the outcome of species interactions. For example, the marine bacteria *Alteromonas macleodii* HOT1A3 enhanced the growth of *Prochlorococcus* MIT9313 with an initial ratio of 1:10 or 1:1 but inhibited it at a higher ratio of 10:1 [19]. In addition, a recent study showed that the initial ratio is very important: pairwise communities with 12 different species were inoculated using different initial ratios [21], which were 1:19 and 19:1 (95% species A to 5% species B and the reciprocal percentages). After 72 h of cultivation, the results demonstrated that 12% of the final pairwise communities displayed legacy dependence on the initial ratio [21]. These results suggested that the social interactions among microbial species are diverse and dynamic, and understanding the initial ratio-associated interactions among species is crucial in predicting microbial community structure and function.

Bacterial interactions include positive, negative, and neutral relationships [11, 22, 23]. Microbes living in the same environment may compete fiercely for space and nutrients [24]; however, they can also cooperate to resist adverse environments [25, 26]. First, metabolic similarity is correlated with bacterial interactions [27]. Previous studies have shown that bacterial interactions can be regulated by niche conditions [28, 29]. It has been found that the spatial structure of a two-species coculture (*Burkholderia* sp. LB400 and *Pseudomonas* sp. B13) can be changed by the carbon sources in the cultural medium two decades earlier [28], showing that the interaction mode of the two strains can be regulated by carbon sources. Therefore, the interplay of initial ratio and niche condition is probably inevitable, and this effect may lead to unpredicted results of cocultures. Unfortunately, most studies usually use only one cultural condition and cannot reveal such an interplay. For example, two *P. fluorescens* strains in multispecies colonies reached a characteristic final ratio of ~1:10, which was independent of the initial ratio ranging from 1:1000 to 1000:1 [20]. Consistent results were obtained for another two cocultured species, *P. aeruginosa* and *Burkholderia cenocepacia* [30], as well as the coculture of *Escherichia coli* and *Salmonella enterica* [31]. All these results indicated that the final community structure is not dependent on the initial ratio in the given cultural condition. By contrast, the medium content or fluidity exhibited stronger influences than the initial ratio on the final ratio of dual species (*Bacillus subtilis* and *Vibrio parahaemolyticus)* cocultures [32]. Although this study implied that niche conditions could overcome the impact of the initial ratio on regulating the community structure, knowledge of how niche conditions interact with the initial ratio and cooperatively regulate the outcome of coculture is still limited.

In this study, we therefore set up a two-species coculture system and cultivated the species with initial ratios of 1:1000–1000:1 in 71 different carbon sources. After identifying the preference of the two species for various carbon sources, the impacts of the carbon source and initial inoculum ratio on the final ratio were investigated. On this basis, the effects of the initial ratio on community function were revealed by comparing the carbon usage profiles of different cultures. To our knowledge, this is the first systematic study to reveal the effect of initial ratios on coculture experiments under diverse culture conditions.

## Materials and methods

### Strains and culture conditions

*E. coli* K-12 (EC) and *P. putida* KT2440 (PP) were stocked in the State Key Laboratory of Agricultural Microbiology (Wuhan, China). All strains were routinely grown in Luria broth (LB) shaking at 180 rpm at 28 °C. *E. coli* and *P. putida* are two of the most common model organisms in the laboratory, and these organisms are widely distributed in various environments, including soil, water, and host-associated niches, in which they likely coexist [33], while the K-12 and KT2440 strains are generally used laboratory strains. Therefore, a two-species coculture system was established with the two bacteria and 71 different carbon sources using a Biolog GEN III microtiter plate [34]. The 71 carbon sources are listed in Table S1. The two-species coculture system had five different initial abundances, two of which were monocultures, and the other three had initial ratios (EC/PP) of 1:1000, 1:1, and 1000:1.

To obtain the initial inoculum, overnight cultures of *E. coli* and *P. putida* (in LB) were centrifuged at 9000 × *g* for 3 min to remove the growth medium (LB). This step was followed by washing three times using Biolog’s IF-0a inoculation fluid and resuspension in IF-0a to a starting colony-forming units of ~1 × 10^7^ (the OD600 of *E. coli* and *P. putida* were ~0.04 and 0.05, respectively). For “1:1”, inoculation was performed by mixing the two components in equal proportion (50 μL per component, the same volumes below); for the “1:1000” coculture, the *E. coli* component was diluted 1000-fold in IF-0a prior to mixing, while the “1000:1” coculture was the reciprocal of the “1:1000”; the “*P. putida*” monoculture was 100 μL of *P. putida*, and the “*E. coli*” monoculture was 100 μL of *E. coli*. After that, inoculums were added into the GEN III plate wells and cultivated at 25 °C, following the manufacturer’s instructions. Assays were performed in triplicate and measured every 4 h for 24 h using Biolog’s MicroStation machine (Biolog, Hayward, CA, USA). The machine collects two absorbance values at 590 and 750 nm. The former value represents a coloration reaction of tetrazolium redox dye, and the latter represents a turbidity index of a culture in a specific carbon source. Since tetrazolium redox dye is reduced during carbon oxidation and results in color generation, the 590 nm absorbance provides a proxy measure for the extent of carbon oxidation in Biolog plate wells. Therefore, we used the 590 nm absorbance value as a measurement of carbon usage efficiency (CUE) in this study. In each experiment, raw values were normalized by subtracting the corresponding value of the negative control in the same plate (A1-zeroed) and then used in subsequent analysis.

### Preference for carbon sources

To distinguish the carbon usage preference of *E. coli* and *P. putida* for the 71 carbon sources, we compared the CUE values of their monocultures grown in each carbon source. Since the CUEs of a species under a carbon source theoretically have a normal distribution, we determined the carbon usage preference of the two species by comparing the mean of three observations. If the mean CUE of *E. coli* in a certain carbon source was significantly higher than the mean CUE of *P. putida* under the same carbon source, the carbon source was considered *E. coli* preferred; if the CUE of *E. coli* in a certain carbon source was significantly lower than that of *P. putida* under the same carbon source, the carbon source was considered *P. putida* preferred. The remaining carbon sources were then considered nonpreferential. In the comparison, a *t*-test was employed, and a *p*-value less than 0.05 was considered significant.

### Coordinate analysis of the carbon usage profiles

To compare the carbon usage profiles of five different cultures, A1-zeroed CUEs (A590) at 24 h, which included 71 (carbon sources) × 5 (groups) × 3 (replicates) observations and formed a 71 × 15 matrix, were used in a principal component analysis with vegan version 2.5–4 [35]. After exploratory inspection of the data, the 71 carbon sources were clustered into three groups using hierarchical clustering with the complete linkage method. Plots were generated using ggplot2 version 3.1 [36] and pheatmap version 1.0.12 (https://CRAN.R-project.org/package=pheatmap). Raw data and analytical codes are provided in the supplementary material (see Data availability).

### Quantifying the relative abundance in coculture using species-specific quantitative PCR

To obtain species-specific primers for *P. putida* and *E. coli*, 16S rRNA gene sequences of each strain were aligned and visualized in BioEdit [37], and primers (Table 1) were picked manually based on the variable regions of 16S rRNA genes with an approximate amplicon size of 300 bp [38]. The specificity of primers was confirmed by PCR with specific and nonspecific templates (Fig. S1). Despite the different sequences, the two sets of primers were demonstrated in later PCR experiments to have similar amplification efficiencies. Genomic DNA was extracted from the 24-h coculture, and the reaction components per 10 μL were 3.5 μL of distilled deionized water, 5 μL of 2× SYBR Green Real-Time PCR master mixture (Applied Biosystems, Foster City, CA, USA), 0.5 μL of 10 μM each primer and 1 μL of genomic DNA. The PCR program was carried out as follows: 95 °C for 5 min, followed by 40 cycles of denaturation at 95 °C for 15 s, annealing at 60 °C for 15 s, and extension and fluorescence reading at 72 °C for 1 min, followed by melting curve analysis between 55 and 95 °C with a heating increment rate of 0.5 °C per 5 s and continuous fluorescence measurement to determine the presence of specific products in the QuantStudio^®^ 6 Flex System (Applied Biosystems, Foster City, CA, USA). For all runs, standard curves were generated by amplification of serial 4× dilutions of the standard plasmid DNA template (in triplicate), with a maximum concentration of 10 ng/μL. The template DNA was thus quantified by using the standard curves (*R*^2^ = 0.99) with QuantStudio™ Real-Time PCR software (version 1.2, Applied Biosystems, Foster City, CA, USA). Similar to the initial ratio (EC/PP), the relative abundances of *E. coli* and *P. putida* in 24-h cultivation were reported as the final ratio of *E. coli* and *P. putida*. With that ratio, proportions could be easily calculated by the equations EC% = ratio/(1+ratio) and PP% = 1/(1+ratio).

**Table 1:** Primers used in this study.

Primer	Sequence (5′-3′)	Note
PP_16Sf	CCTTGCTGTTTTGACGTTAC	*P. putida*-specific forward primer
PP_16Sr	ATCTCAAGGATTCCAACGGC	*P. putida*-specific reverse primer
EC_16Sf	CCTTTGCTCATTGACGTTAC	*E. coli*-specific forward primer
EC_16Sr	GCCTCAAGGGCACAACCTCC	*E. coli*-specific reverse primer

### Defining bacterial interactions in coculture

**Fig. 1:**
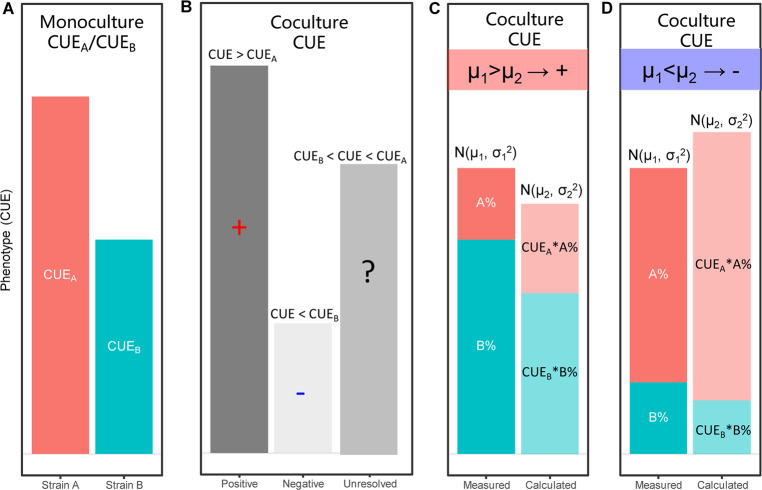
The interaction model used in this study. **a** The phenotypes (CUE) of strain A and strain B in monoculture are CUE_A_ and CUE_B_, respectively. They are assumed not equal, and CUE_A_ is higher than CUE_B_. **b** When strains A and B were cocultivated, the coculture CUE could generally fall into three aspects: (1) positive, if coculture CUE > CUE_A_; (2) negative, if the CUE < CUE_B_; and (3) unresolved, if the CUE was between CUE_A_ and CUE_B_. In this study, the unresolved mode was further diagnosed by an additional hypothesis test method to reveal the interaction mode more accurately. **c**, **d** The examples of positive and negative modes of interaction derived from “unresolved” in (**b**). A% and B% are the relative abundances of strains A and B in coculture, respectively. The *x*-axis represents the measured CUE and calculated CUE, which have normal distributions with the mean μ_1_ and μ_2_, respectively. By comparing μ_1_ and μ_2_, we can further obtain the interaction mode. μ_1_ > μ_2_ indicates a positive interaction, and μ_1_ < μ_2_ indicates a negative interaction (see Methods).

For coculture systems, the overall CUE is related to the CUE of the two species included and their proportion in the system (Fig. 1). In the simplest case, if the total CUE is higher than the maximum CUE in the monocultures, it is a positive interaction; if the total CUE is lower than the minimum CUE in the monocultures, it is a negative interaction (Fig. 1b). However, in fact, the CUE of the coculture is unlikely to exhibit these cases but is normally between the maximum and the minimum; therefore, we need to consider the proportion of the two species as well (Fig. 1c, d). Based on this, we propose the following theoretical model.

The definition of bacterial coculture interaction was derived from a well-defined classification scheme through a hypothesis test approach [39]. However, we have appended several improvements to the original scheme. In general, we assessed the effect of coculture on the CUE based on the monoculture CUEs. As we know the relative abundance of each species in the coculture, we first calculated the theoretical CUE of a coculture by summing the monoculture CUE and its proportion in coculture (Fig. 1c, d). The measured CUE and calculated CUE were assumed to have normal distributions, with means of μ_1_ and μ_2_. We compared the difference of μ_1_ and μ_2_ by hypothesis *t*-test and selected 0.05 as the significance level. First, consider the hypothesis H_0_: μ_1_ < μ_2_ and compute the *p*-value *P*_neg_. If *P*_neg_ < 0.05, the observation is unlikely under the hypothesis and is thus an indication of a positive effect (Fig. 1c). Second, if *P*_neg_ ≥ 0.05, consider the hypothesis H_0_:μ_1_ < μ_2_, and compute the *p*-value *P*_pos_. If *P*_pos_ < 0.05, the observation was classified as a negative effect (Fig. 1d). The rest of the classifications have an unresolved effect. This method considers the species abundance in coculture and thus improves the resolution and accuracy of the test compared with that of the original definition [39]. In this model, relative abundance is critical in determining bacterial interactions. For instance, some cocultures with the same CUE can lead to different results due to abundance differences (Fig. 1c, d).

### Statistics

All statistical analyses were conducted by using R software (version 3.6, http://www.R-project.org). To meet assumptions of normality and homogeneity of variance, the quantities of *E. coli* and *P. putida* measured by qPCR were log10 transformed. Multiple linear regression was applied to normalized datasets to reveal the associations between key parameters, including CUE, the populations of *E. coli* and *P. putida*, carbon preference and initial ratios (see Data availability). Two-group comparisons were performed using Student’s *t*-test, and multiple group comparisons were performed using one-way ANOVA and a post hoc test to find variations between different groups, unless otherwise stated. The *p*-value in multiple comparisons was adjusted by the “BH” method. For all statistical analyses, a *p*-value (or adjusted *p*-value if applicable) less than 0.05 was considered statistically significant.

## Results

### Final ratio of cocultures

**Fig. 2:**
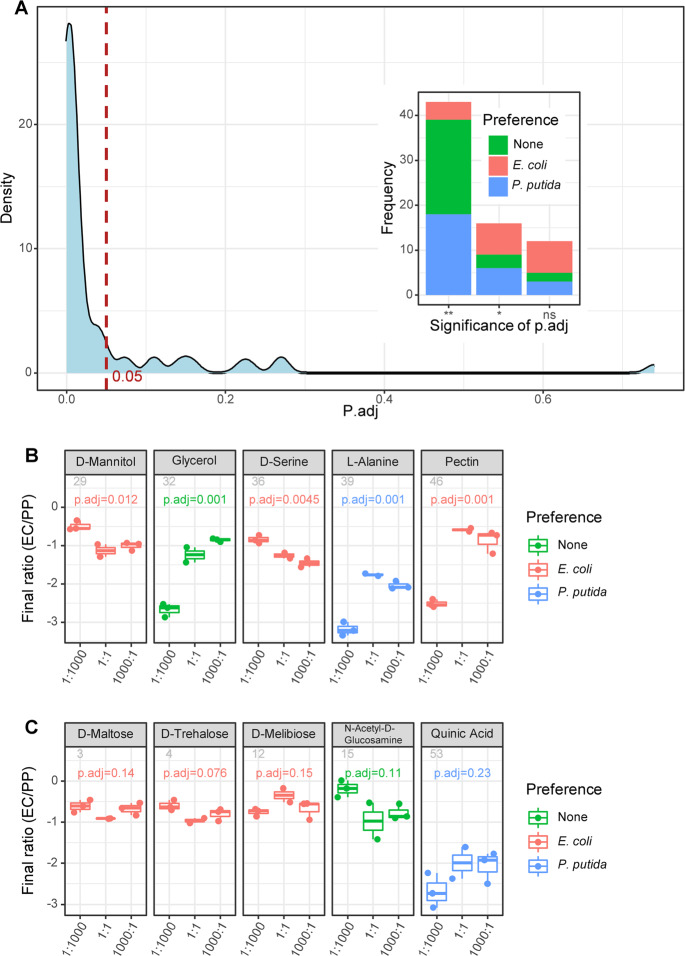
Final ratios of cocultures. **a** The density of the adjusted *p*-value (ANOVA) in testing whether the final ratios of the three cocultures are different in all carbon sources. The *x*-axis represents the adjusted *p*-value (BH method), and the vertical line indicates the position of the *p*-value cutoff (0.05). Inset: The barplot shows the frequency of significance of the adjusted *p*-value (**p < 0.01, *p < 0.05, ns, *p* ≥ 0.05). In the barplot, frequencies were colored by carbon preference. Five examples for significant results (**b**) or nonsignificant results (**c**) of final ratios are given. In (**b**, **c**), the *y*-axis represents the log10-transformed final ratio.

*P. putida*, rather than *E. coli*, takes the major proportion of cocultures in overall all the carbon sources. Hence, almost all of the final ratios, which are the ratio of *E. coli* quantity and *P. putida* quantity in 24-h cultivation, are less than 1, and the median of them is 0.036, with the first and third quartiles being 0.0047 and 0.09, respectively. The final ratios of the three cocultures differed significantly in 59 carbon sources (ANOVA, *p* < 0.05, Fig. 2a), including 43 carbon sources with a *p*-value < 0.01. However, there were still 12 carbon sources that had nonsignificant results (Fig. 2a). Although the final ratios differed in most of the cocultures, the correlation of the final ratio and initial ratio was weak, as the highest final ratios of cocultures were observed for the “1:1000” coculture (d-mannitol and d-serine), “1000:1” coculture (glycerol) and “1:1” coculture (pectin) (Fig. 2b). In addition, the carbon preference did not correlate with the final ratios (Fig. 2). Both nonsignificant and significant results occurred with *E. coli-* and *P. putida*-preferred carbon sources. Interestingly, we found that the frequency of *E. coli*-preferred carbon sources was higher than that of *P. putida*-preferred among the nonsignificant results (Fig. 2, see Fig. S2 for complete results of all carbon sources).

**Fig. 3:**
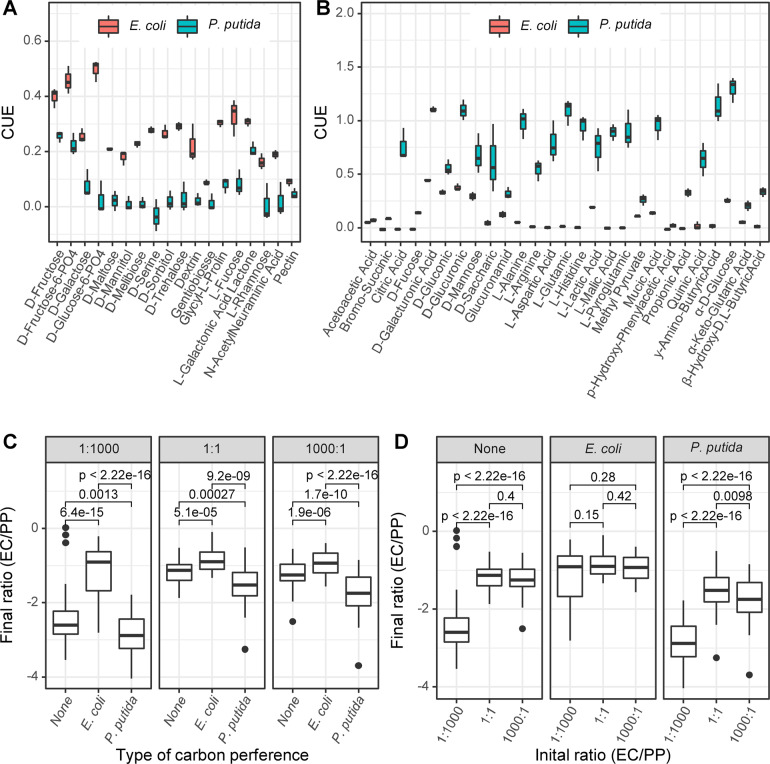
Carbon preferences. Among 71 different carbon sources, there were 18 *E. coli*-preferred (**a**) carbon sources and 27 *P. putida*-preferred (**b**) carbon sources. The *x*-axis is the name of carbon sources, and the *y*-axis is the CUE of monocultures. **c** Comparison of the final ratios of a given coculture in different types of carbon sources defined by preference. The *x*-axis is the type of carbon preference, and the *y*-axis is the final ratio (log10 transformed). Left: the “1:1000” coculture; middle, the “1:1” coculture; right: the “1000:1” coculture. **d** Comparison of the final ratios between three cocultures in a given type of carbon source. The *x*-axis indicates coculture initial ratios, and the *y*-axis is the final ratio (log10 transformed). Left: in nonpreferred carbon sources (none); middle: in *E. coli*-preferred carbon sources; right: in *P. putida*-preferred carbon sources. The *p*-values given on the top of bracket in (**c**, **d**) were calculated by pairwise Wilcoxon rank sum test.

Eighteen carbon sources were preferred by *E. coli* (Fig. 3a), 27 carbon sources were preferred by *P. putida* (Fig. 3b), and the remaining 26 carbon sources showed no preference between the two bacteria. When grown in preferred carbon sources, the monoculture of *E. coli* has a significantly higher CUE than that of *P. putida*, and vice versa (Fig. 3a, b). We subsequently quantified the final ratio of the three cocultures in every carbon source and compared the results among the different types of carbon sources grouped by preference. In all three cocultures, the highest final ratio presented with *E. coli*-preferred carbon sources, and the lowest final ratio presented with *P. putida*-preferred carbon sources (Fig. 3c). Pairwise *p*-values (Wilcoxon rank sum test) between different types of carbon preferences were all significant. This result emphasizes that growing in a preferred carbon source is beneficial for increasing the relative abundance of either *E. coli* or *P. putida* in cocultures. Interestingly, the final ratio of the three cocultures was similar for the combined results in *E. coli*-preferred carbon sources (Fig. 3d), suggesting that *E. coli* in the 1:1000 and 1:1 cocultures exhibited a comparable proportion to that in the 1000:1 cocultures. This result is consistent with the multiple linear regression result, which showed that growing in *E. coli*-preferred carbon source is the most important positive influence factor on the final ratio (*p* < 0.001, see Data availability). By contrast, the “1:1000” coculture had a significantly lower final ratio in both nonpreferred and *P. putida*-preferred carbon sources than the “1:1” and “1000:1” cocultures (Fig. 3d). Taken together, these results indicated that in addition to the initial ratio, the available nutrients greatly influence the final ratio of cocultures, suggesting that whether the assembly of the coculture is history dependent can be overcome by culture conditions.

### The initial ratio regulates the metabolic capacities of cocultures

The CUE profile with the 71 different carbon sources was measured as an overall metabolic capacity for monocultures and cocultures. It reflects the comprehensive metabolic capacity of a species or community under diverse nutrient conditions. While comparing the CUE profiles between different groups (Fig. S3A), we found that the median CUE of the “1:1” coculture had the greatest value (0.46), followed by that of the “1000:1” coculture (0.352), *P. putida* monoculture (0.091), “1:1000” coculture (0.086), and *E. coli* monoculture (0.056) groups. Specifically, the median CUE of the *P. putida* monoculture was approximately twofold higher than that of the *E. coli* monoculture, reflecting that the overall metabolic activity of the *P. putida* monoculture is higher than that of the *E. coli* monoculture. Notably, the “1:1” and “1000:1” cocultures but not the “1:1000” coculture both have higher metabolic capacity than the *P. putida* monoculture. The coexistence of *P. putida* and *E. coli* enhanced the overall metabolic capacities in the two cocultures but weakened the capacity in the other coculture.

**Fig. 4:**
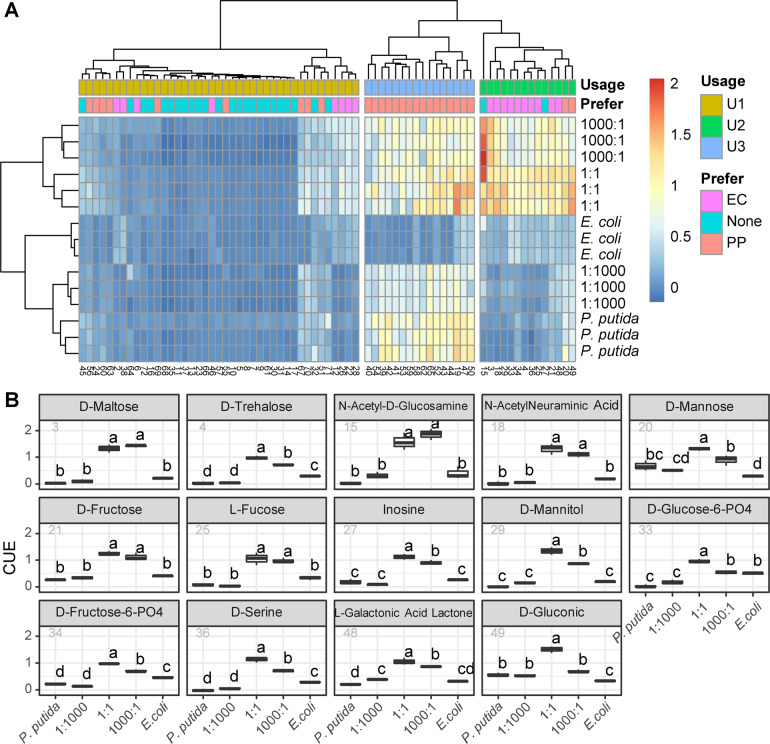
The initial ratio regulates carbon usage profiles of cocultures. **a** Clustering of carbon sources by usage groups. In the heatmap, the type of carbon source is indicated by bars on the top, the carbon ID is indicated on the bottom, and experimental replicates are given on the right. The legend bar indicates the range of CUE values. **b** The CUE of mono- and cocultures with 14 U2 carbon sources (from left to right, top to bottom). The *x*-axis indicates culture conditions, and the *y*-axis indicates CUE. ANOVA and Tukey’s multiple comparisons test were performed. The text on the boxplot indicates whether significant variances were observed between different cultures.

A clustered heatmap showing the differences across 71 carbon sources was created with two monocultures and three cocultures clustered into three groups, and carbon sources clustered into three groups: U1, U2, and U3 (Fig. 4a). The first culture cluster had only the *E. coli* monoculture, the second cluster had the *P. putida* monoculture and the “1:1000” coculture, while the third cluster had the “1:1” and “1000:1” cocultures (see also Fig. S3B for the principal component analysis of CUE profiles). In carbon source clusters, U1 has 41 carbon sources, and the CUE values in this group are generally low across all groups, indicating that these carbon sources are mostly difficult to utilize. U1 mainly includes 24 nonpreferred carbon sources, followed by nine *P. putida*-preferred and eight *E. coli*-preferred carbon sources. U2 has 14 carbon sources, and the CUE values of the “1:1” and “1000:1” cultures are much higher than those of the other cultures. U2 has ten *E. coli*-preferred carbon sources, two nonpreferred ones and two *P. putida-*preferred carbon sources. U3 has 16 carbon sources, and the CUE values are high for all the groups except the *E. coli* monoculture. All U3 carbon sources are *P. putida* preferred. Obviously, the CUEs of 14 U2 carbon sources are the main factor causing significant differences between the “1:1”/“1000:1” cultures and the others. They are basically unable to be efficiently utilized by both the *P. putida* monoculture, the *E. coli* monoculture, or the “1:1000” coculture. However, the “1:1” and “1000:1” cocultures obtained a high capacity of using 14 extra carbon sources when inoculated with the specific initial ratios.

Figure 4b shows the CUE of cultures with these 14 U2 carbon sources (see Fig. S4 for complete results of all carbon sources). The cocultivation of *E. coli* and *P. putida* was not enough for the community to gain access to U2 carbon sources, as the correct initial ratios were needed. For example, the CUE of the “1:1000” coculture had no significant variances with either the *P. putida* or *E. coli* monocultures in 7 U2 carbon sources, which are d-maltose, N-acetyl-D-glucosamine, N-acetylneuraminic acid, d-fructose, l-fucose, inosine, and d-mannitol (Fig. 4b). In addition, the CUEs of the “1:1” and “1000:1” cocultures were similar and were significantly higher than those of the other groups (*p* < 0.05) in most cases, if not all.

We used multiple linear regression to explore the correlations among CUE, initial ratio and carbon sources, which were further clustered by carbon preference or usage (Table S1, Fig. 4). The regression model results showed that 68% of the dependent variable variation could be explained based on adjusted R-squared (0.6782, *p* < 2.2e−16). The factors that significantly influence CUE were, from strong to weak, carbon usage group, initial ratio, and carbon preference. In addition, when the other parameters were controlled, either increasing the initial ratio from 1:1 to 1000:1 or decreasing the initial ratio from 1:1 to 1:1000 will significantly lower CUE (see Data availability).

Taken together, these results indicated that the initial ratio is able to regulate the metabolic capacity of cocultures under various niche conditions.

### The initial ratio and carbon sources cooperatively regulate bacterial interactions

**Fig. 5:**
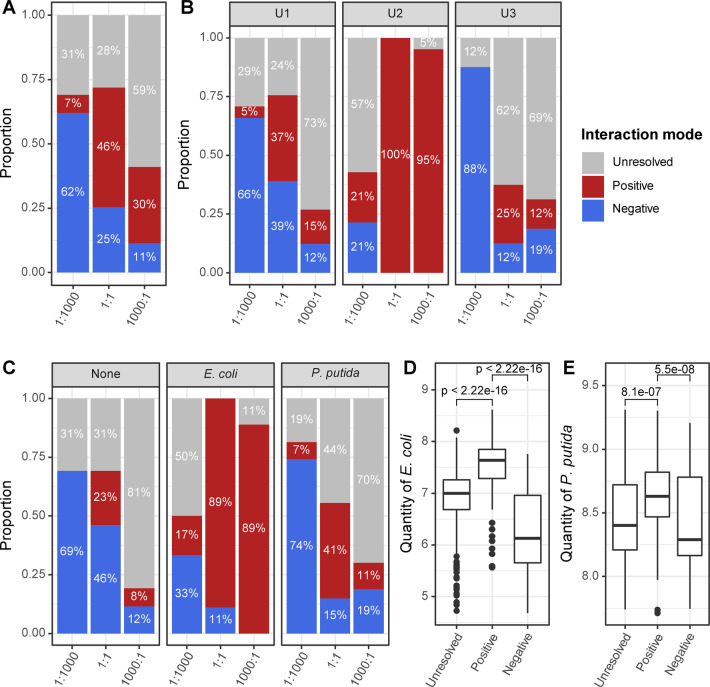
Bacterial interactions in cocultures. **a** Overall comparison of the interaction mode between the three cocultures with all carbon sources. The results were, furthermore, summarized into three parts by the carbon usage groups (**b**) and preference (**c**). Comparisons of *E. coli* (**d**) and *P. putida* (**e**) quantities in different bacterial interaction modes are shown as well. For **a**–**c**, the proportions were colored by interaction mode. For **d**–**e**, the *x*-axis indicates the interaction mode, and the *y*-axis indicates the log10-transformed bacterial quantities of *E. coli* and *P. putida*, respectively.

We evaluated the interaction mode of cocultures in utilizing carbon sources, as defined in Methods (Fig. 1). We found that the “1:1000” cocultures had 62% negative interactions and only 7% positive interactions; the “1:1” cocultures had 25% negative interactions and 46% positive interactions; and the “1000:1” cocultures had 11% negative interactions and 30% positive interactions (Fig. 5a). The two species in the “1:1” and “1000:1” cocultures were more likely to utilize carbon sources synergistically than the “1:1000” coculture.

To clarify the influence of the type of carbon source, we divided the result into three parts according to the carbon usage groups (Fig. 5b) and preference (Fig. 5c). The results showed that the “1:1” cocultures had the most positive interactions, while the “1:1000” cocultures had the most negative interactions, regardless of the type of carbon source (Fig. 5b, c). In U1 carbon sources, 37% of the “1:1” cocultures showed a positive interaction, followed by the “1000:1” and “1:1000” cocultures (Fig. 5b, left panel). In U2 carbon sources, almost all of the “1:1” and “1000:1” cocultures had positive interactions (Fig. 5b, middle panel). This result is consistent with the intrinsic property of the 14 carbon sources in U2, as they can be effectively utilized only by the “1:1” and “1000:1” cocultures (Fig. 4). In U3 carbon sources, we found that no positive effect was found in the “1:1000” cocultures (Fig. 5b, right panel). This result means that the presence of *E. coli* in the “1:1000” coculture may not have any positive effect on utilizing these carbon sources in cocultures, which is consistent with the observation that all U3 carbon sources can be utilized by *P. putida* (Fig. 4a). Furthermore, more positive effects were found in *E. coli*-preferred carbon sources (Fig. 5c, middle panel) than in *P. putida*-preferred carbon sources (Fig. 5c, right panel). In addition, the result for nonpreferred carbon sources was quite similar to that for U1 carbon sources (Fig. 5c, left panel). These results showed that the initial ratio had a great impact on bacterial interaction and that the characteristics of carbon sources can modulate the effect of the initial ratio on bacterial interaction. Although there are some exceptions (Fig. S5), it seems that positive interaction is more likely to be established when the species populations are comparable.

In addition, we found that the establishment of a positive interaction is beneficial to increase the population of both *E. coli* and *P. putida* in cocultures, while a negative interaction significantly lowers their quantity compared with that in the situation of unresolved interactions (Fig. 5d, e). Furthermore, the increment of the *E. coli* median quantity in positive cocultures was approximately fivefold greater than that in unresolved cocultures (Fig. 5d) and was much greater than that of *P. putida* in positive cocultures (Fig. 5e). Positive interaction enhanced the carbon source utilization capacity, while increased assimilation of carbon sources led to an increase in the total cell number in cocultures.

## Discussion

The current study investigated the effect of initial inoculation ratios on the final function and structure of bacterial cocultures. We found that the initial ratio can influence the structure and, more importantly, the function and the bacterial interaction of a community. Our results suggested that the interaction between the two species is not stable and preset but can be changed by the initial inoculation ratio and niche conditions.

**Fig. 6:**
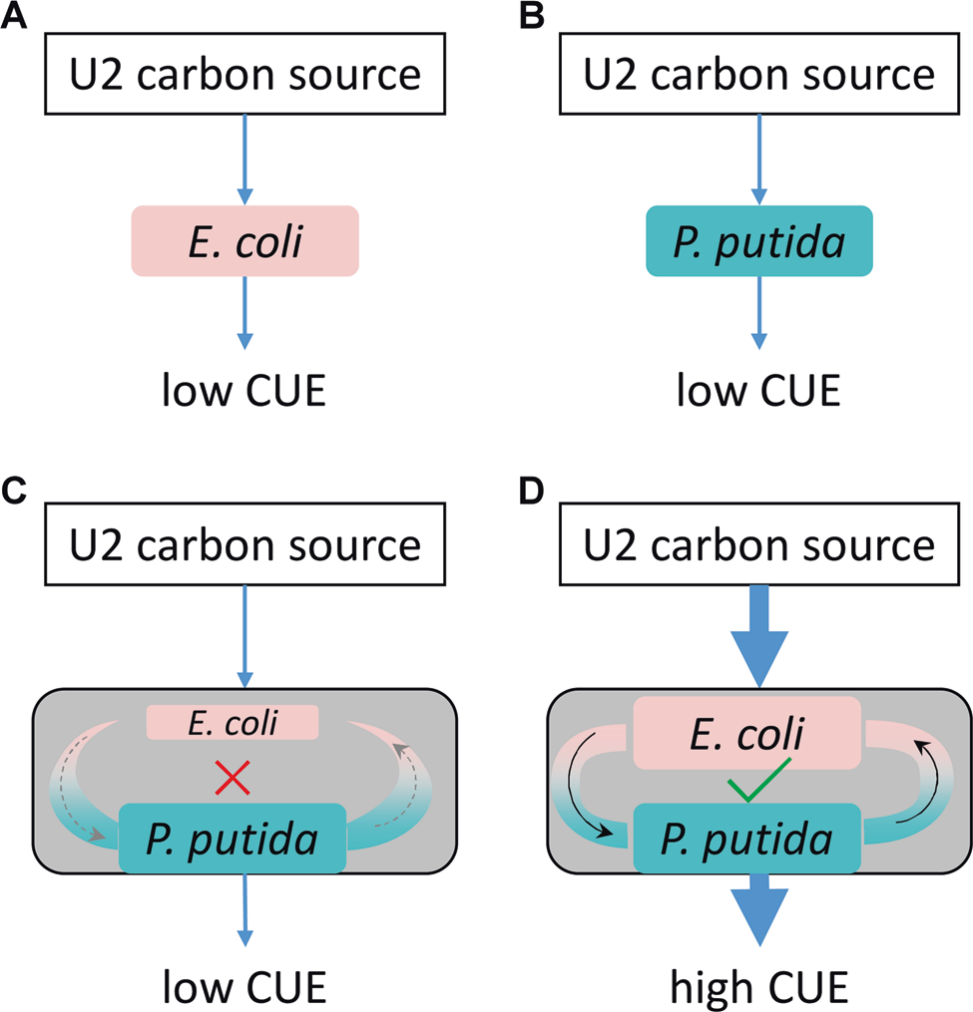
Metabolic coupling in utilizing U2 carbon sources. The *E. coli* monoculture (**a**) and *P. putida* monoculture (**b**) have only low efficiency in utilizing U2 carbon sources. In a coculture that has a smaller *E. coli* population, metabolic coupling is difficult to establish, as *E. coli* is the bottleneck restricting the flow of metabolites (**c**). When *E. coli* has an equivalent population to *P. putida*, metabolic flow can be established and lead to high CUE for U2 carbon sources (**d**).

To date, we generally assumed that species interaction comes from the nature of the involved species, and the interaction is treated as an intrinsic property between species. Negative interactions are attributed to resource competition and the secretion of antibiotics [40, 41], while positive interactions are attributed to metabolic coupling [42, 43], cross-feeding [3, 44], auxotrophies [26], and so on. Therefore, the type of bacterial interactions [17] together with the bacterial metabolic capacity [45] and bacterial carbon usage preference [12] are important parameters in predicting the structure of a complex community. However, the current study revealed that the interaction is regulated by the relative abundance of the involved species, and it is not an intrinsic but an emergent property between species. When *E. coli* was less abundant than *P. putida*, more negative interactions were observed, while more positive interactions were observed when *E. coli* was equal to or more abundant than *P. putida* (Fig. 6). This situation will inevitably increase the unpredictability of community behavior.

We found that the “1:1” and “1000:1” cocultures showed synergistic cooperation in utilizing 14 U2 carbon sources, thereby greatly improving the CUE (Fig. 6). The key may be that the two species establish a metabolic coupling circuit or cross-feeding by integrating metabolic capacities. The genomic basis for establishing such a metabolic coupling was also present in the “1:1000” coculture; however, it failed to result in a successful metabolic coupling pathway involving both species. A possible explanation for this observation could be that the 14 U2 carbon sources could be metabolized only by a joint effort of both *E. coli* and *P. putida*, but the population of *E. coli* in the “1:1000” coculture was too small to provide enough resources for a co-metabolic flow, thus failing to establish the co-metabolism pathway (Fig. 6c). In contrast, the other two cocultures successfully established the co-metabolic pathway (Fig. 6d), which greatly improved the CUE of U2 carbon sources. Notably, the presence of *E. coli* in the “1:1000” coculture even had an adverse effect on the use of U2 carbon sources, accounting for approximately one-fifth of the combinations (Fig. 5b, middle panel). This result suggests that such an influence of the initial ratio is continuous rather than just a legacy effect.

The impact of the initial ratio on the final ratio had a trade-off for carbon sources. For example, the final ratios were not significantly different among the three cocultures when grown in *E. coli*-preferred carbon sources (Fig. 3d). Therefore, whether the assembly of two-species cocultures is history dependent also depends on the available nutrients. Under natural conditions, communities with similar structures may also move toward different outcomes due to differences in niche conditions. Correspondingly, communities with different structures may also converge due to the same niche condition [46]. Only U2 carbon sources induced the metabolic coupling of *E. coli* and *P. putida*, while the other carbon sources did not have such potential. We believe that this effect could be an example of the check and balance between bacterial adaptation capacity and numerous niche conditions.

The simplest approach for establishing a coculture system is to use a low-diversity model community from well-known, well-characterized laboratory strains [47]. *E. coli* and *P. putida* are ubiquitous microorganisms that inhabit soil, water, animal tissue, humans, and plant surfaces [33]. Both are important components of the biosphere; however, the optimal growth temperatures of *E. coli* and *P. putida* are ~40 and 30 °C, respectively [48, 49]. It should be noted that the culture temperature in our assays was more suitable for the growth of *P. putida*; thus, *P. putida* might be highly abundant in most of the coculture systems. However, free-living bacteria are frequently exposed to niche shifts and nonoptimal growth conditions. A prior study has also shown that *E. coli* can survive under temperature, oxygen, and pH stress conditions [50]. In this study, we have shown that the proportion of *E. coli* was highly improved in preferred carbon sources, implying that culture conditions can overcome the growth limitation of temperature for *E. coli*. Therefore, we believe that the choice of temperature has little effect on the conclusion of this study.

## Supplementary information

Supplementary materials

## Data Availability

Raw data from Biolog and qPCR experiments, together with the analytical codes in R, have been uploaded to a GitHub repository (http://github.com/gaospecial/ratio).

## References

[1] Nai C, Meyer V (2018). From axenic to mixed cultures: technological advances accelerating a paradigm shift in microbiology. Trends Microbiol.

[2] Powers MJ, Sanabria-Valentín E, Bowers AA, Shank EA (2015). Inhibition of cell differentiation in *Bacillus subtilis* by *Pseudomonas protegens*. J Bacteriol.

[3] Sztajer H, Szafranski SP, Tomasch J, Reck M, Nimtz M, Rohde M (2014). Cross-feeding and interkingdom communication in dual-species biofilms of *Streptococcus mutans* and *Candida albicans*. ISME J.

[4] Trejo-Hernández A, Andrade-Domínguez A, Hernández M, Encarnación S (2014). Interspecies competition triggers virulence and mutability in *Candida albicans*–*Pseudomonas aeruginosa* mixed biofilms. ISME J.

[5] Garbeva P, Silby MW, Raaijmakers JM, Levy SB, de Boer W (2011). Transcriptional and antagonistic responses of *Pseudomonas fluorescens* Pf0-1 to phylogenetically different bacterial competitors. ISME J.

[6] Yoshida S, Ogawa N, Fujii T, Tsushima S (2009). Enhanced biofilm formation and 3-chlorobenzoate degrading activity by the bacterial consortium of *Burkholderia* sp. NK8 and *Pseudomonas aeruginosa* PAO1. J Appl Microbiol.

[7] Beliaev AS, Romine MF, Serres M, Bernstein HC, Linggi BE, Markillie LM (2014). Inference of interactions in cyanobacterial–heterotrophic co-cultures via transcriptome sequencing. ISME J.

[8] Ren D, Madsen JS, Sørensen SJ, Burmølle M (2015). High prevalence of biofilm synergy among bacterial soil isolates in cocultures indicates bacterial interspecific cooperation. ISME J.

[9] Al-Shabib NA, Husain FM, Ahmad I, Khan MS, Khan RA, Khan JM (2017). Rutin inhibits mono and multi-species biofilm formation by foodborne drug resistant *Escherichia coli* and *Staphylococcus aureus*. Food Control.

[10] Liu W, Jacquiod S, Brejnrod A, Russel J, Burmølle M, Sørensen SJ (2019). Deciphering links between bacterial interactions and spatial organization in multispecies biofilms. ISME J.

[11] Li M, Wei Z, Wang J, Jousset A, Friman VP, Xu Y (2019). Facilitation promotes invasions in plant-associated microbial communities. Ecol Lett.

[12] Goyal A, Dubinkina V, Maslov S (2018). Multiple stable states in microbial communities explained by the stable marriage problem. ISME J.

[13] Zhalnina K, Louie KB, Hao Z, Mansoori N, da Rocha UN, Shi S (2018). Dynamic root exudate chemistry and microbial substrate preferences drive patterns in rhizosphere microbial community assembly. Nat Microbiol.

[14] Faust K (2019). Microbial consortium design benefits from metabolic modeling. Trends Biotechnol.

[15] Liang J, Bai Y, Men Y, Qu J (2017). Microbe–microbe interactions trigger Mn(II)-oxidizing gene expression. ISME J.

[16] Xu X, Zarecki R, Medina S, Ofaim S, Liu X, Chen C (2019). Modeling microbial communities from atrazine contaminated soils promotes the development of biostimulation solutions. ISME J.

[17] Kong W, Meldgin DR, Collins JJ, Lu T (2018). Designing microbial consortia with defined social interactions. Nat Chem Biol.

[18] Kelvin Lee KW, Hoong Yam JK, Mukherjee M, Periasamy S, Steinberg PD, Kjelleberg S (2016). Interspecific diversity reduces and functionally substitutes for intraspecific variation in biofilm communities. ISME J.

[19] Aharonovich D, Sher D (2016). Transcriptional response of *Prochlorococcus* to co-culture with a marine *Alteromonas*: differences between strains and the involvement of putative infochemicals. ISME J.

[20] Kim W, Levy SB, Foster KR (2016). Rapid radiation in bacteria leads to a division of labour. Nat Commun.

[21] Venturelli OS, Carr AV, Fisher G, Hsu RH, Lau R, Bowen BP (2018). Deciphering microbial interactions in synthetic human gut microbiome communities. Mol Syst Biol.

[22] Little AE, Robinson CJ, Peterson SB, Raffa KF, Handelsman J (2008). Rules of engagement: interspecies interactions that regulate microbial communities. Annu Rev Microbiol.

[23] West SA, Diggle SP, Buckling A, Gardner A, Griffin AS (2007). The social lives of microbes. Annu Rev Ecol Evol Syst.

[24] Foster KR, Bell T (2012). Competition, not cooperation, dominates interactions among culturable microbial species. Curr Biol.

[25] Lee KWK, Periasamy S, Mukherjee M, Xie C, Kjelleberg S, Rice SA (2014). Biofilm development and enhanced stress resistance of a model, mixed-species community biofilm. ISME J.

[26] Zengler K, Zaramela LS (2018). The social network of microorganisms—how auxotrophies shape complex communities. Nat Rev Microbiol.

[27] Russel J, Røder HL, Madsen JS, Burmølle M, Sørensen SJ (2017). Antagonism correlates with metabolic similarity in diverse bacteria. Proc Natl Acad Sci.

[28] Nielsen AT, Tolker-Nielsen T, Barken KB, Molin S (2000). Role of commensal relationships on the spatial structure of a surface-attached microbial consortium. Environ Microbiol.

[29] Hansen SK, Rainey PB, Haagensen JAJ, Molin S (2007). Evolution of species interactions in a biofilm community. Nature.

[30] Leinweber A, Fredrik Inglis R, Kümmerli R (2017). Cheating fosters species co-existence in well-mixed bacterial communities. ISME J.

[31] Fazzino L, Anisman J, Chacón JM, Heineman RH, Harcombe WR (2020). Lytic bacteriophage have diverse indirect effects in a synthetic cross-feeding community. ISME J.

[32] Gao CH, Zhang M, Wu Y, Huang Q, Cai P (2019). Divergent influence to a pathogen invader by resident bacteria with different social interactions. Micro Ecol.

[33] Molina-Santiago C, Udaondo Z, Cordero BF, Ramos JL (2017). Interspecies cross-talk between co-cultured *Pseudomonas putida* and *Escherichia coli*. Environ Microbiol Rep.

[34] Mallon CA, Le Roux X, van Doorn GS, Dini-Andreote F, Poly F, Salles JF (2018). The impact of failure: unsuccessful bacterial invasions steer the soil microbial community away from the invader’s niche. ISME J.

[35] Oksanen J, Kindt R, Legendre P, O’Hara B, Stevens MHH, Oksanen MJ (2007). The vegan package. Community Ecol Package.

[36] Wickham H. Ggplot2: elegant graphics for data analysis. New York, USA: Springer Publishing Company; 2009.

[37] Hall T (1999). BioEdit: a user-friendly biological sequence alignment editor and analysis program for Windows 95/98/NT. Nucleic Acids Symp Ser.

[38] Ren D, Madsen JS, de la Cruz-Perera CI, Bergmark L, Sørensen SJ, Burmølle M (2014). High-throughput screening of multispecies biofilm formation and quantitative PCR-based assessment of individual species proportions, useful for exploring interspecific bacterial interactions. Micro Ecol.

[39] Madsen JS, Røder HL, Russel J, Sørensen H, Burmølle M, Sørensen SJ (2016). Coexistence facilitates interspecific biofilm formation in complex microbial communities. Environ Microbiol.

[40] Hibbing ME, Fuqua C, Parsek MR, Peterson SB (2010). Bacterial competition: surviving and thriving in the microbial jungle. Nat Rev Microbiol.

[41] Szamosvári D, Rütschlin S, Böttcher T (2018). From pirates and killers: does metabolite diversity drive bacterial competition?. Org Biomol Chem.

[42] Burmølle M, Ren D, Bjarnsholt T, Sørensen SJ (2014). Interactions in multispecies biofilms: do they actually matter?. Trends Microbiol.

[43] Hansen LB, Ren D, Burmølle M, Sørensen SJ (2017). Distinct gene expression profile of *Xanthomonas retroflexus* engaged in synergistic multispecies biofilm formation. ISME J.

[44] Solden LM, Naas AE, Roux S, Daly RA, Collins WB, Nicora CD (2018). Interspecies cross-feeding orchestrates carbon degradation in the rumen ecosystem. Nat Microbiol.

[45] Freilich S, Zarecki R, Eilam O, Segal ES, Henry CS, Kupiec M (2011). Competitive and cooperative metabolic interactions in bacterial communities. Nat Commun.

[46] Goldford JE, Lu N, Bajić D, Estrela S, Tikhonov M, Sanchez-Gorostiaga A (2018). Emergent simplicity in microbial community assembly. Science.

[47] Røder HL, Sørensen SJ, Burmølle M (2016). Studying bacterial multispecies biofilms: where to start?. Trends Microbiol.

[48] Munna MS, Zeba Z, Noor R (2015). Influence of temperature on the growth of *Pseudomonas putida*. Stamford J Microbiol.

[49] Gonthier A, Guerin-Faublee V, Tilly B, Delignette-Muller ML (2001). Optimal growth temperature of O157 and non-O157 *Escherichia coli* strains. Lett Appl Microbiol.

[50] Van Elsas JD, Semenov AV, Costa R, Trevors JT (2011). Survival of *Escherichia coli* in the environment: fundamental and public health aspects. ISME J.

