# Supplementary material for: The initial inoculation ratio regulates bacterial coculture interactions and metabolic capacity
Source: ISME J. 2020 Sep 4;15(1):29–40. doi: 10.1038/s41396-020-00751-7 (PMC7852870; doi:10.1038/s41396-020-00751-7)
Supplement: Supplementary file 1 — Supplementary materials [file 41396_2020_751_MOESM1_ESM.pdf]

Supplementary files of

The initial inoculation ratio regulates bacterial coculture interactions and metabolic capacity

Chun-Hui Gao[1#], Hui Cao[1#], Peng Cai[1*], Søren J. Sørensen[2]

[1]State Key Laboratory of Agricultural Microbiology, College of Resources and Environment, Huazhong Agricultural University, Wuhan 430070, China. [2]Section of Microbiology, Department of Biology, University of Copenhagen, Copenhagen, Denmark.[*]Corresponding author: Peng Cai, cp@mail.hzau.edu.cn

[#]These authors contributed equally to this article.

The authors declare no competing financial interests.

Table S1 List of 71 carbon sources

Id	Name	Usage[1]	Prefer[2]
**2**	Dextrin	U1	EC
**3**	D-Maltose	U2	EC
**4**	D-Trehalose	U2	EC
**5**	D-Cellobiose	U1	None
**6**	Gentiobiosse	U1	EC
**7**	Sucrose	U1	None
**8**	D-Turanose	U1	None
**9**	Stachyose	U1	None
**10**	D-Raffinose	U1	None
**11**	α-D-Lactose	U1	None
**12**	D-Melibiose	U1	EC
**13**	β-Methyl-D-Glucoside	U1	None
**14**	D-Salicin	U1	None
**15**	N-Acetyl-D-Glucosamine	U2	None
**16**	N-Acetyl-β-Dmannosamine	U1	None
**17**	N-Acetyl-D-Galactosamine	U1	None
**18**	N-AcetylNeuraminic Acid	U2	EC
**19**	α-D-Glucose	U3	PP
**20**	D-Mannose	U2	PP
**21**	D-Fructose	U2	EC
**22**	D-Galactose	U1	EC
**23**	3-MethylGlucose	U1	None
**24**	D-Fucose	U1	PP
**25**	L-Fucose	U2	EC

**26**	L-Rhamnose	U1	EC
**27**	Inosine	U2	None
**28**	D-Sorbitol	U1	EC
**29**	D-Mannitol	U2	EC
**30**	D-Arabitol	U1	None
**31**	myo-Inositol	U1	None
**32**	Glycerol	U1	None
**33**	D-Glucose-6-PO4	U2	EC
**34**	D-Fructose-6-PO4	U2	EC
**35**	D-Aspartic Acid	U1	None
**36**	D-Serine	U2	EC
**37**	Gelatin	U1	None
**38**	Glycyl-L-Prolin	U1	EC
**39**	L-Alanine	U3	PP
**40**	L-Arginine	U3	PP
**41**	L-Aspartic Acid	U3	PP
**42**	L-Glutamic	U3	PP
**43**	L-Histidine	U3	PP
**44**	L-Pyroglutamic	U3	PP
**45**	L-Serine	U1	None
**46**	Pectin	U1	EC
**47**	D-Galacturonic Acid	U3	PP
**48**	L-Galactonic Acid Lactone	U2	EC
**49**	D-Gluconic	U2	PP
**50**	D-Glucuronic	U3	PP
**51**	Glucuronamid	U1	PP
**52**	Mucic Acid	U3	PP

**53**	Quinic Acid	U3	PP
**54**	D-Saccharic	U3	PP
**55**	p-Hydroxy-Phenylacetic Acid	U1	PP
**56**	Methyl Pyruvate	U1	PP
**57**	D-Lactic Acid Methyl Ester	U1	None
**58**	L-Lactic Acid	U3	PP
**59**	Citric Acid	U3	PP
**60**	α-Keto-Glutaric Acid	U1	PP
**61**	D-Malic Acid	U1	None
**62**	L-Malic Acid	U3	PP
**63**	Bromo-Succinic	U1	PP
**64**	Tween 40	U1	None
**65**	y-Amino-ButryricAcid	U3	PP
**66**	α-Hydroxy-ButyricAcid	U1	None
**67**	β-Hydroxy-D,L-ButyricAcid	U1	PP
**68**	α-Keto-ButyricAcid	U1	None
**69**	Acetoacetic Acid	U1	PP
**70**	Propionic Acid	U1	PP
**71**	Acetic Acid	U1	None
**72**	Formic Acid	U1	None

Note: [1] Carbon usage groups were determined by hierarchical clustering of CUE profiles of all cultures (see Figure 4 for more information). [2] Carbon preferences were determined by comparing the CUE of monocultures as follows (see also Figure 3). EC, *Escherichia coli* K-12 preferred carbon source; PP, *Pseudomonas putida* KT2440 preferred carbon source; None: no preference.

Figure S1

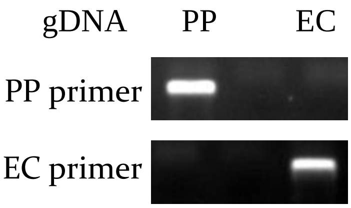

Figure S1. Specificity of species-specific primers. The PCR experiments were performed with *E. coli* and *P. putida* specific primers (left) and their genomic DNA (top), respectively. EC, *Escherichia coli* K-12; PP, *Pseudomonas putida* KT2440.

Figure S2

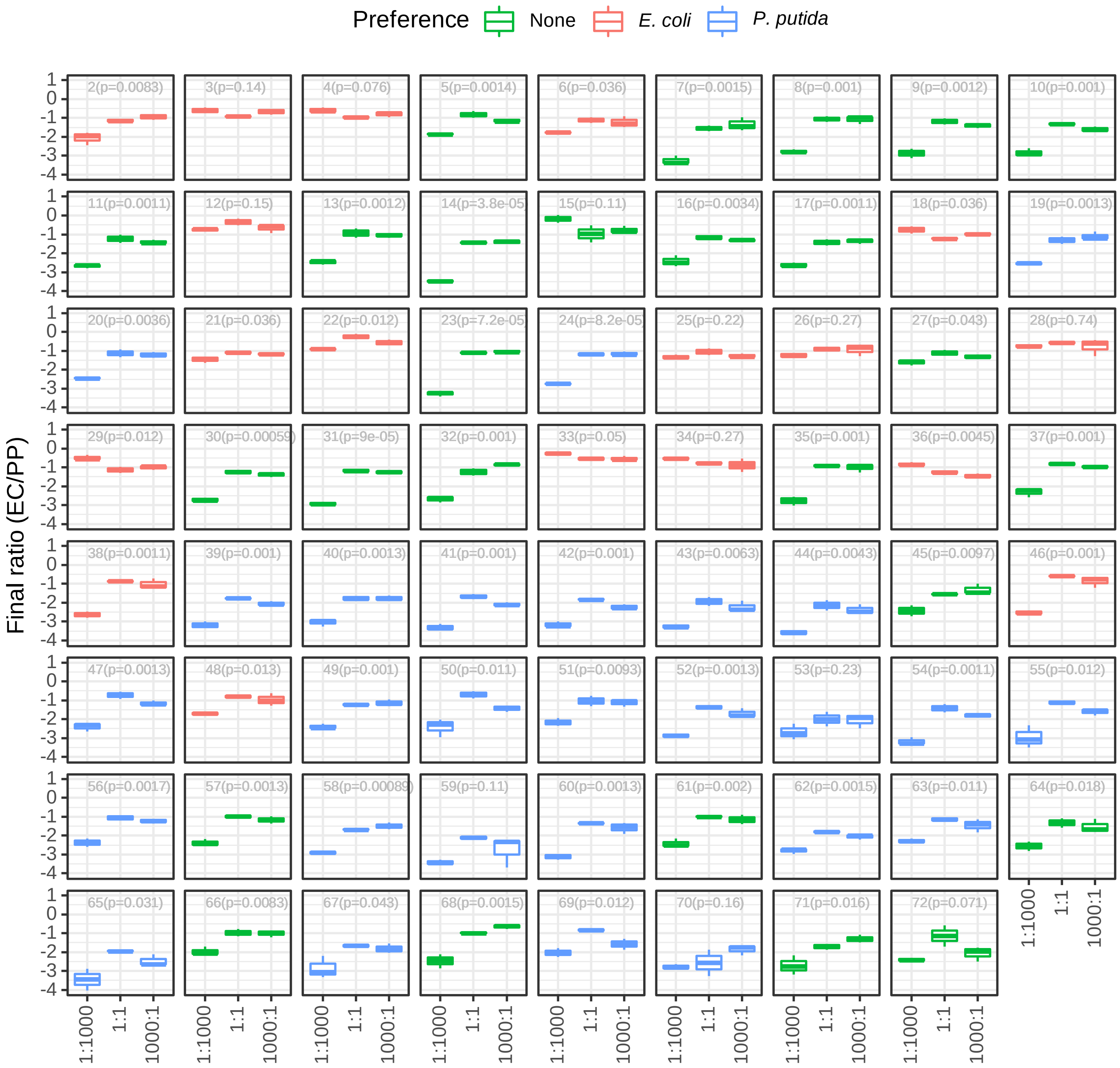

Figure S2. Final ratio of all cocultures in 71 carbon sources (left to right, top to down, carbon id from 2 to 72). The adjusted p-value (ANOVA) were showed on the top of each subplot. The x-axis indicates coculture initial ratios (*E. coli*/*P. putida*), and the y-axis is the final ratio (log10-transformed). Green, none preferred carbon sources; Red, *E. coli* preferred carbon sources; Blue, *P. putida* preferred carbon sources.

Figure S3

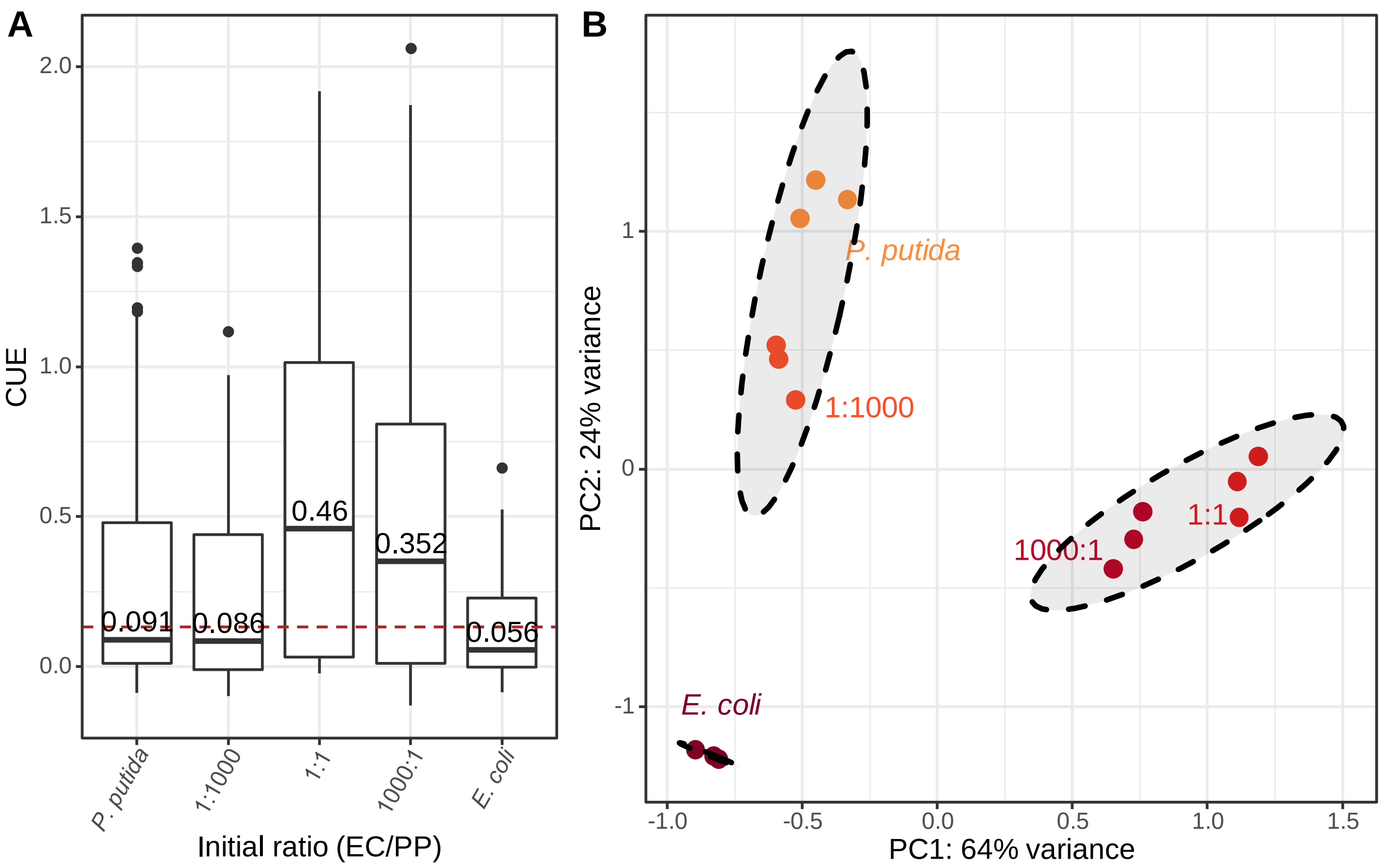

Figure S3. The carbon usage profiles of two monocultures (*E.coli* and *P. putida*) and three cocultures (1:1000, 1:1, and 1000:1). (A) boxplot. Number shows the median of each culture, and horizontal line shows the mean of all cultures. (B) PCA analysis of carbon usage profiles. Ellipses represent the 95% confidence interval of clustering.

Figure S4

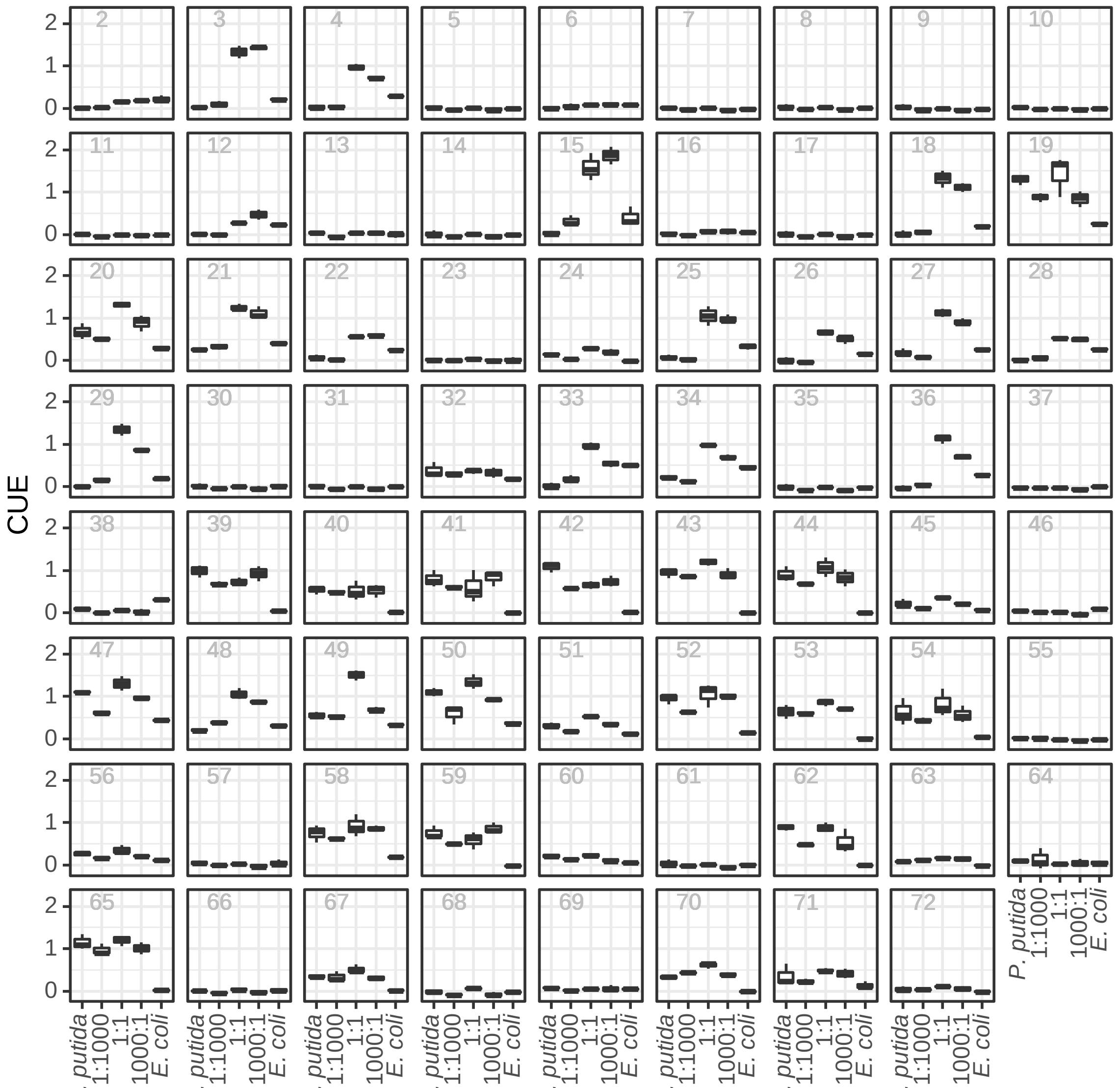

Figure S4. The CUE of mono- and cocultures in 71 carbon sources (left to right, top to down, carbon id from 2 to 72). The x-axis indicates culture conditions (there are a total of 5 culture conditions: *P. putida*, the “1:1000” coculture ; the “1:1” coculture; the “1000:1” coculture and *E. coli*), and the y-axis indicates CUE.

Figure S5

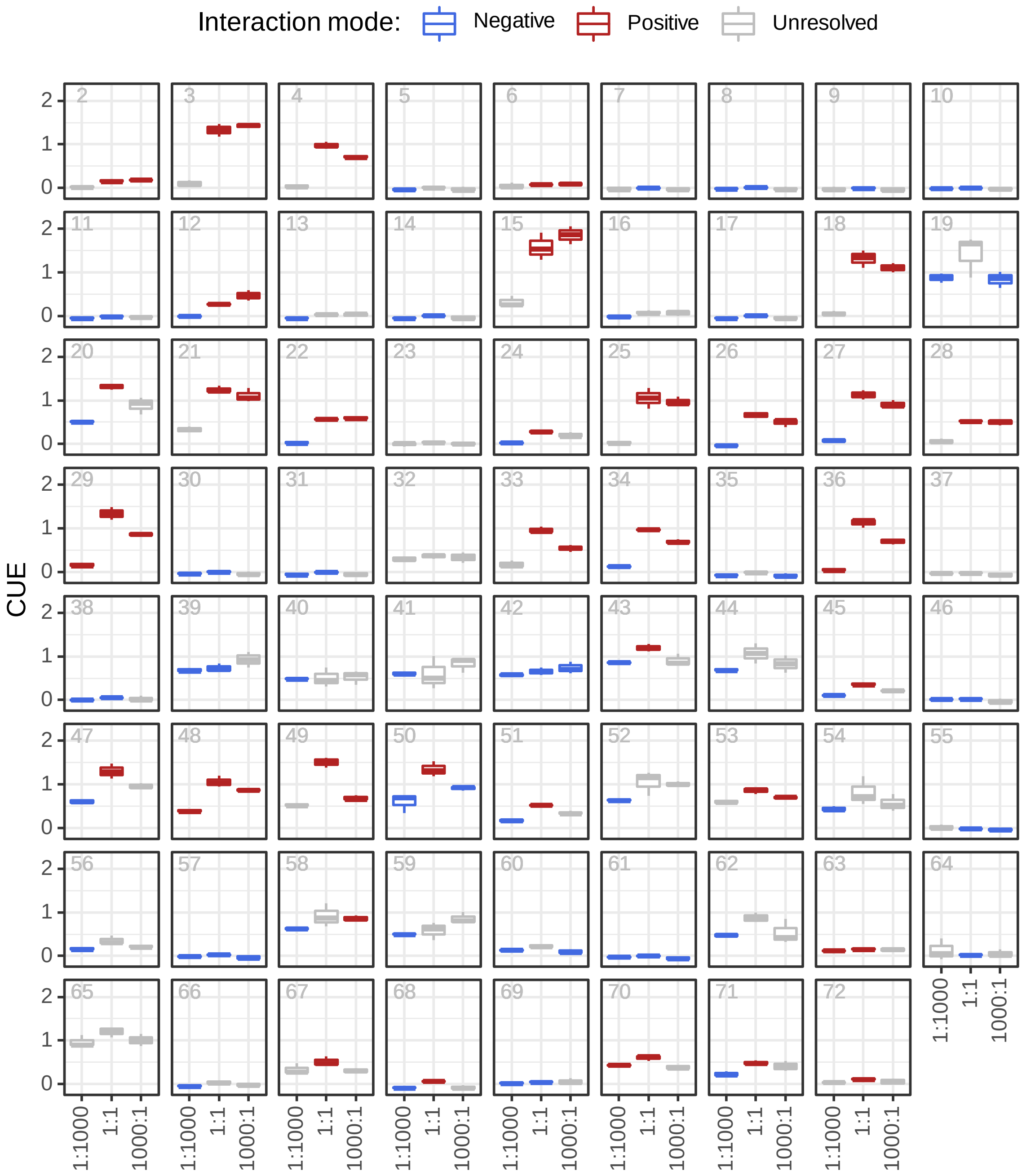

Figure S5. The interaction mode of all combinations in 71 carbon sources (left to right, top to down, carbon id from 2 to 72). The x-axis indicates coculture initial ratios (*E. coli*/*P. putida*), and the y-axis indicates CUE. Blue, negative interaction; Red, positive interaction; Gray, unresolved interaction.
